# Effects of Thermal Demagnetization in Air on the Microstructure and Organic Contamination of NdFeB Magnets

**DOI:** 10.3390/ma17225528

**Published:** 2024-11-13

**Authors:** Laura Grau, Rosario Moreno López, Pierre Kubelka, Fabian Burkhardt, Tomaž Tomše, Spomenka Kobe, Carlo Burkhardt

**Affiliations:** 1 Institute for Precious and Technology Metals (STI), Pforzheim University, Tiefenbronner Str. 65, 75175 Pforzheim, Germany; rosariomoreno.bcn@gmail.com (R.M.L.); pierre.kubelka@hs-pforzheim.de (P.K.); tomaz.tomse@ijs.si (T.T.); carlo.burkhardt@hs-pforzheim.de (C.B.); 2 Jožef Stefan International Postgraduate School, Jamova Cesta 39, 1000 Ljubljana, Slovenia; spomenka.kobe@ijs.si; 3 Department for Nanostructured Materials (K7), Jožef Stefan Institute, Jamova Cesta 39, 1000 Ljubljana, Slovenia; fabian.burkhardt@ijs.si

**Keywords:** thermal demagnetization, NdFeB recycling, NdFeB demagnetization

## Abstract

Demagnetization is an essential step for the demounting and safe handling of end-of-life NdFeB. Thermal demagnetization in air is a straightforward option to demount adhesive-fixed or segmented magnets. However, this process is suspected to increase the uptake of contaminants like O, C and Zn from coatings and adhesives, potentially degrading the recyclate quality. This study tests the effects of thermal demagnetization in air at 400 °C for 15 to 240 min on variously coated samples with different initial oxidation levels. Furthermore, the possible reversal of the contaminant uptake is explored. Samples with low previous oxidation levels showed significant uptake in oxygen with a minimal diffusion depth, while the uptake depended on the used coating. The best protectiveness was achieved with NiCuNi with an increase in oxygen of only around 30%. Epoxy (up to ~130% O uptake) and Zn coatings (up to ~80% O uptake) disintegrated during the treatment and offered less protection but still made a difference compared to uncoated samples (up to ~220% O uptake). Samples with high initial oxidation levels show no clear tendency towards further oxygen uptake and the carbon uptake is generally low, likely due to contemporary epoxy coatings featuring a passivation underneath as a barrier layer. Zn infiltration, which carried organic debris, was observed. Short demagnetization times proved to be favorable for limiting the depth of the diffusing contaminants. Mechanical coating removal after thermal demagnetization in air can mitigate the contaminant uptake, producing clean, recyclable end-of-life material.

## 1. Introduction

The recycling of metallic end-of-life (EOL) scrap, which includes permanent magnets (PM), poses significant challenges due to the tendency of ferrous scrap to stick together. This issue has led to the recommendation of avoiding PM in favor of design for recycling [1]. However, there are instances, such as in motors used in battery electric vehicles (BEV), handheld power tools and others, where avoiding PM may not be technically favorable: the lower energy efficiency and torque density of PM-free motors [2,3] would have to be compensated by larger batteries, lower power or shorter usage times. This underscores the need for a solution that allows for the recycling of PM, especially high-performance NdFeB magnets made from critical raw materials.

An environmentally friendly recycling method is the hydrogen processing of magnetic scrap (HPMS) with a subsequent re-sintering of the produced NdFeB powder [4]. A comprehensive overview of hydrogen-based recycling methods and routes is given in Burkhardt et al. [5]. For this, the majority of scraps needs to be demounted and, thus, demagnetized before the treatment, as the presence of strong magnetic fields will complicate the removal. A rare exception are scraps suitable for in situ treatments [6,7], as the HPMS process produces demagnetized powders [8].

While various methods are available for demagnetization, thermal demagnetization stands out for its simplicity and applicability. In comparison to electromagnetic demagnetization with a decaying field, known as degaussing, thermal demagnetization is a highly accessible and rather fail-proof method, as long as a sufficient temperature above the Curie temperature is reached. Significantly higher temperatures and long hold times may degrade the magnet and coating materials, though. Conversely, degaussing requires a tailored tool to generate targeted reverse fields of sufficient strength, and it may lead to residual magnetization [9]. Such tools may also lack the flexibility recyclers require with diverse scrap.

In contrast, thermal demagnetization only requires a furnace that can accommodate the specimens and maintain a temperature above the specific Curie temperature of the magnetic scrap [9], and, if necessary, the degradation temperature of present adhesives. This is an added benefit of thermal demagnetization: the decomposition of adhesives before demounting further reduces the forces to remove the magnets from the assembly. Using an ambient atmosphere accelerates the degradation of polymers compared to an inert atmosphere, where the carbon-rich contents are pyrolyzed, leaving carbon-rich char residue [10]. Not requiring inert process gas and an infrastructure to introduce them is beneficial for cost-efficiency and the environment. On the other hand, the uptake of organic contaminants like oxygen and carbon at elevated temperatures is assumed, which would deteriorate the magnetic properties of recycled magnets made from the affected scrap, since the formation of oxides and carbides hinders the formation of a continuous grain boundary Nd-rich phase [11,12]. Also, the elevated levels of carbon present during sintering lead to the disproportionation of the hard magnetic phase due to the binding of dissolved Nd, resulting in the formation of α-iron, further reducing the coercivity [13]. This especially applies to the intended direct recycling method HPMS, as here, the powder is meant to be used as is without any kind of chemical reduction treatment, underlining the necessity to limit and monitor the contamination uptake.

The application of coatings and the process of passivation are common measures to protect permanent magnets from corrosion and general contaminant uptake while also offering further benefits depending on the type of coating. For example, epoxy resin-based coatings are common due to their electrically isolative nature to limit eddy currents. Other common coatings are Ni-based like NiCuNi multilayered coatings and Zn-based coatings. These three coating types are most commonly found in end-of-life scrap together with combination coatings like Zn–epoxy.

In this paper, the effects of a combined treatment to thermally demagnetize and to decompose fixation adhesives and organic coatings are tested in the context of recyclability via hydrogen processing and re-sintering. The EOL magnets with different coatings treated by the combined treatment are analyzed in SEM for microstructure and coating integrity and in ONH and CS before and after. This serves to quantify, understand and mitigate the contaminant uptake of C and O. Additionally, the uptake of O and Zn near the magnet surface or coating are monitored in SEM-EDS. This study is limited to the contamination of common coatings induced by thermal demagnetization in air.

## 2. Materials and Methods

### 2.1. Materials

For the investigation, EOL magnets, as well as scrap NdFeB magnets available from different waste streams, were screened. Samples were chosen to both represent common NdFeB coatings, as well as EOL magnets with a low initial oxygen content, which are hereby classified as contamination samples (lc), (defined as <1000 ppm O) and those with higher initial oxygen contents, hereby classified as higher contamination (hc) (defined as >1000 ppm O). The high contamination samples are expected to be mostly saturated with oxides, even though they have a high variation of initial oxygen contents before treatment.

One low initial contamination and one higher initial contamination sample type were taken for each of the following coatings: nickel–copper–nickel (NiCuNi), zinc or zinc–epoxy (Zn or ZnEp) and epoxy (Ep), as well as uncoated magnets (uc). Each sample group comprises individual magnets received from the same source (e.g., the same manufacturer or the same EOL electric machine). In contrast, the origin of the waste streams of the individual magnets varied.

The samples were selected from the available range of end-of-life and production scrap magnets acquired in several research projects to reflect the aforementioned criteria and to have similar dimensions.

**Figure 1:**
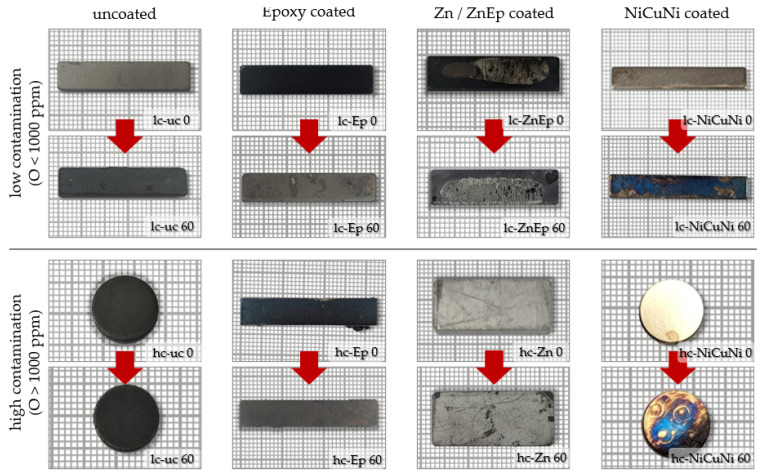
All samples used, before and after demagnetization. The change in visual aspects by demagnetization (signified by the arrows between the images) is shown. For demagnetization and for removing organic coatings and glues, the samples were treated at 400 °C in air for 60 min.

The particularly suitable lc-Ep sample group (lowest initial contamination, low initial variance before treatment, favorable geometry) was used to create uncoated magnets lc-uc for testing by removing the coating by sandblasting prior to the treatment. Due to sample preparation constraints, this test’s range of usable magnets was limited to smaller-dimension magnets with low thicknesses below 4 mm. The magnets are shown before and after treatment in Figure 1.

### 2.2. Thermal Demagnetization

Thermal demagnetization was carried out in a Heraeus thermicon P muffle furnace at 400 °C in an ambient atmosphere for a minimum of 15 min and a maximum of 240 min.

The temperature was chosen as it is sufficient in both thermally demagnetizing and decomposing present organic resins.

The base alloy of NdFeB has a Curie temperature *T_c_* of 312 °C [14], but it can be enhanced by doping the alloy with heavy rare earth and transition metals, like cobalt and aluminum [15,16].

The decomposition of basic epoxy resins begins at approximately 200 °C, and nearly complete mass loss is achieved at around 400 °C in air [17]. In previous thermal demagnetization trials with temperatures of up to 400 °C, all adhesive connections present in the samples were separable and the majority of them disintegrated. Exceptions were segmented magnets, where the exposure of the adhesive layer to air is limited and special heat-resistant adhesives with suitable additives were used.

To produce the lc-uc samples from lc-Ep samples, sandblasting was performed using a corundum abrasive at 6 bar compressed air in an HGH 1100 I sandblasting chamber, fully removing the epoxy coating and the passivation layer below.

### 2.3. Preparation and Analysis Methods

Epoxy coatings were carefully ground off with #500 grit corundum sandpaper to determine the samples’ oxygen and carbon content. This step is crucial as the coatings predominantly consist of organic compounds, which would interfere with the accuracy of the results. Metallic coatings were not removed. To reduce the influence of the order of measurements, all samples were placed in air for 24 h ± 2 h after thermal demagnetization treatment.

Oxygen values were determined using the LECO ONH836 (LECO Corporation, St. Joseph, MI, USA). Carbon values were measured using the LECO CS744 (LECO Corporation, St. Joseph, MI, USA). The samples were not cut to defined dimensions in order to avoid contamination with water or organic cutting fluids, which would falsify the O and C content. Therefore, samples for both measurements were produced by impact fragmenting the treated magnets and choosing fragments in the weight range of 0.35–0.70 g. Due to the apparent relevance of surface effects, only nearly prismatic samples, where approximately half of the sample surface is covered by the coating or the exposed surface area, were considered to keep the treated surface to fragment volume ratio as consistent as possible.

For optical observation and EDS analysis, a Hitachi Flex-SEM 1000 II (Hitachi High-Tech, Tokyo, Japan) with a W-cathode and an Oxford Instruments micsF+ x-stream-2 EDS (Oxford Instruments, Wycombe, UK) as well as the field emission FEI Nova nanoSEM (FEI Company, Hillsboro, OR, USA) with an Oxford Instruments ULTIM MAX 65 EDS (Oxford Instruments, Wycombe, UK) were used.

## 3. Results and Discussion

### 3.1. Visual Aspect

Figure 1 shows samples before and after a 60 min thermal demagnetization treatment at 400 °C. The uncoated sample lc-uc significantly darkened. The uncoated sample hc-uc had a darker appearance beforehand, likely due to prior surface oxidation, but it also darkened further.

The epoxy disintegrated progressively during thermal demagnetization. Therefore, the original protective function of the coating is impaired, which should be kept in mind for the further processing after demagnetization. The demagnetized magnets should be immediately processed further or stored in protective gas.

By itself, thermal demagnetization is not a suitable means to fully remove epoxy coatings. The decomposition of the organic resins leaves a charred residue as the compounds are not fully volatilized. After 60 min, such residues can be fully removed with a dry cloth. Below the epoxy, the passivation is visible; see Figure 1 lc-Ep 60 and hc-Ep 60 as opposed to lc-uc 60 and hc-uc 60 without a passivation layer.

The NiCuNi coatings are tarnished and turn blue- and orange-colored.

The Zn–epoxy coating of lc-ZnEp shows a dark gray color, and while the coating flakes are burnt off, it appears as if the lower layer has darkened. The Zn coating of hc-ZnEp appears thinner and dark areas emerge where the coating is scratched.

### 3.2. Microstructure

The microstructure is monitored close to the coating or the exposed magnet surface. Bulk images from the middle of the samples are omitted as no significant differences between the treated and non-treated samples are seen. When the magnets are observed on the Scanning Electron Microscope (SEM) in combination with EDS before and after treatment, the most prominent detectable changes are the diffusion of contaminants and the formation of cracks between the grains.

**Figure 2:**
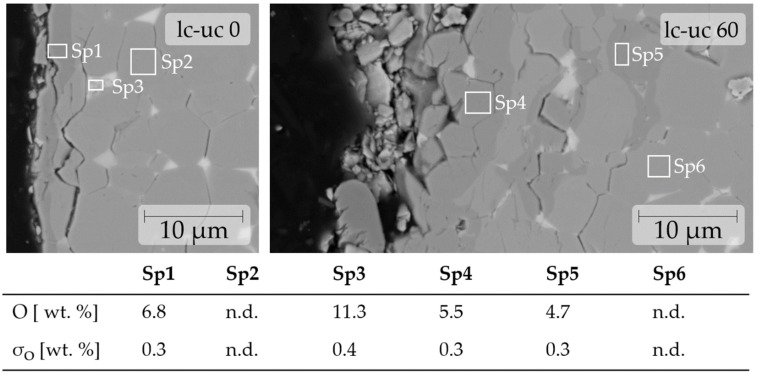
The diffusion of oxygen in lc-uc during demagnetization is visualized by SEM. EDS measurements should only be interpreted for O detection and rough quantification. Hitachi FlexSEM 2500× BSE, 20 kV.

In Figure 2, enhanced oxygen contents can be seen in the matrix grains propagating along the grain boundaries. Unlike the visible change seen for the oxygen uptake, the carbon diffusion itself was not possible to monitor on SEM-EDS due to the loss of sensitivity of the energy dispersive x-ray spectroscopy (EDS) sensors and the BSE contrast with the decreasing atomic number and a low proportion of carbon in the material. Generally, SEM-EDS only yields qualitative results for oxygen too, as more elaborate techniques (e.g., TEM-EELS) would be necessary to reliably quantify the local oxygen content.

**Figure 3:**
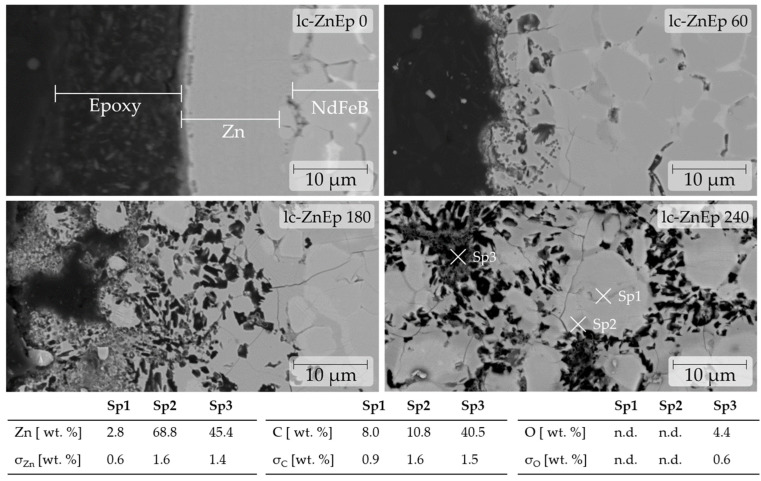
The diffusion of Zn and epoxy residues is visualized by SEM. EDS measurements should only be interpreted as a qualitative analysis. Hitachi Flex SEM 2500× BSE, 20 kV for lc-ZnEp 0–lc-ZnEp 180. FEI NanoSEM 3500× BSE with Oxford Instruments Ultim Max 65 EDS detector for lc-ZnEp 240, with higher resolution and count rates in EDS.

An exception for the detection of carbon are large spots with high carbon content, which can be spotted easily in backscattered electron imaging (BSE). This was observed in the treated ZnEp samples. Figure 3 shows the deep diffusion of C and O particles, possibly pieces of epoxy in grain boundaries infiltrated by a dark gray phase, identified as Zn-rich, replacing the Nd-rich phase. While the diffusion of Zn along the grain boundaries of NdFeB has been reported before [18], it is also seen that the infiltrating Zn-rich phase appears to act as a carrier to loose organic residues.

With the increasing demagnetization time, the Zn–epoxy infiltration progresses deeper into the material and pushes apart the grains, which is documented by the SEM images seen in Figure 3. In the first hour, the infiltration depth is within several micrometers but reaches up to 150 µm depth at some spots after 240 min. Overall, the Zn layer appears to be disintegrated even though the demagnetization is carried out below the melting point of 419.5 °C [19].

### 3.3. Quantification of Oxygen and Carbon Uptake

**Figure 4:**
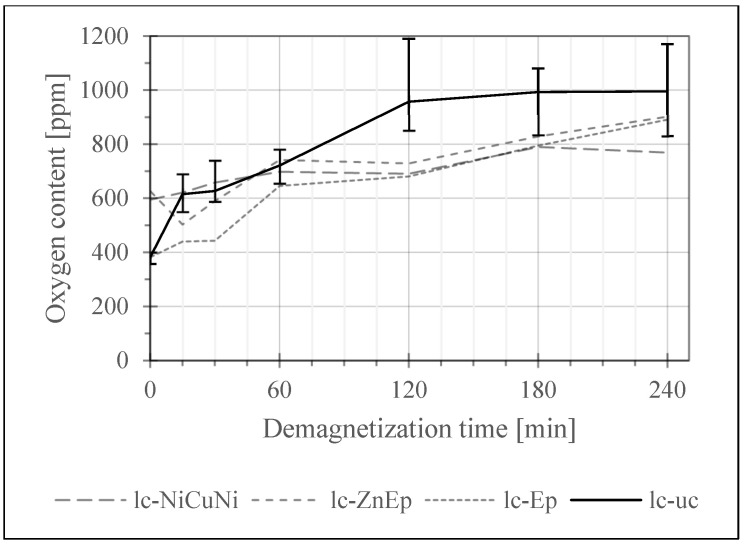
Development of the oxygen content of low contamination samples over demagnetization time. Measured with Leco ONH836. Single measurements were taken for all samples except for the most sensitive sample, lc-uc, which was measured ten times to illustrate the expected maximum range of oxygen values at each time point.

**Figure 5:**
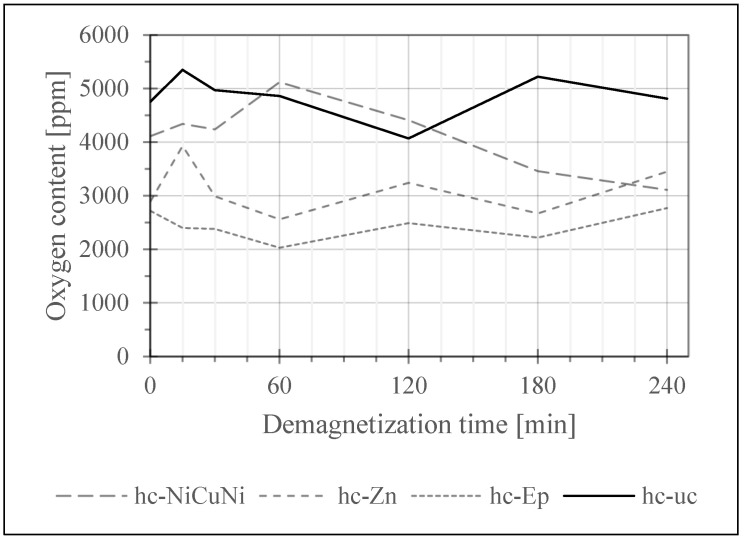
Development of the oxygen content of high contamination samples over demagnetization time. Measured with Leco ONH836.

**Figure 6:**
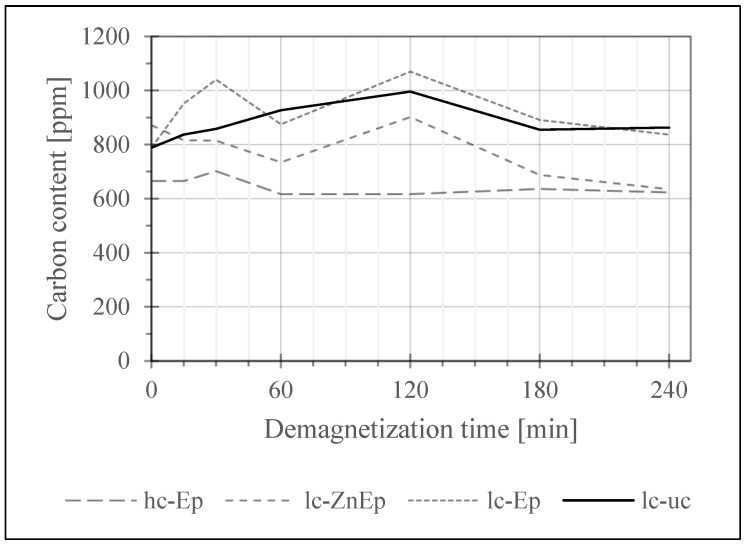
Development of the carbon content of carbon-relevant samples over demagnetization time. Measured with Leco CS744.

In a first screening test, the samples of each magnet type were exposed to the demagnetization treatment at 400 °C in air for 15 min, 30 min, 60 min, 120 min, 180 min and 240 min with one sample for each time and sample group. This test aimed to observe if there are differences in the oxygen and carbon uptake depending on the initial contamination and coating. The results are seen in Figure 4, Figure 5 and Figure 6.

To establish the maximum possible error within the constraint of limited numbers of samples, the most sensitive and most readily available samples (lc-uc) which also showed very consistent initial values were tested ten times per demagnetization hold time. The error bars of the lc-uc graph in Figure 4 represent the minimum and maximum O values measured for each time, giving an idea about the variance in oxygen levels caused by the demagnetization treatment.

Figure 4 shows the development of the oxygen content over time for low contamination (lc) samples, defined as samples with an initial oxygen content below 1000 ppm. Here, the oxygen content of all thermally demagnetized samples increases over time. As expected, the increase is highest for the uncoated lc-uc sample, where the oxygen content at 240 min increased by ~160% compared to the initial value. After 120 min, there is barely any further uptake measured, likely due to saturation of the surface with oxides.

For the lc-Ep sample, the increase in this timeframe was ~130%. Both samples had a very low starting oxygen content, which may have contributed to the substantial increase. For the lc-ZnEp, the increase was ~45%, but ~80% from the minimum value, as a dip in the curve is seen at 15 min, which is likely attributed to the considerable variation of values as seen in the error calculated for lc-uc. The lowest increase in oxygen content was seen in the lc-NiCuNi sample at ~30%. This shows that while there is a tendency for the magnets to pick up oxygen, the uptake differs based on the used coating and its individual protectivity. It is further indicated that the saturation of the surface layers with oxides hinders further uptake.

Figure 5 shows the same measurements for high contamination samples (hc samples, defined as samples with an initial oxygen content above 1000 ppm) which do not show a tendency to increase. This is attributed to the high variance of initial values compared to the likely very low oxygen uptake due to the samples being mostly saturated with oxides already.

The change in carbon content is further monitored in Figure 6. Here, only the epoxy or Zn–epoxy-coated samples and, for comparison, the uncoated lc-uc sample are shown. The Zn- and NiCuNi-coated samples are omitted as they do not contain carbon. Again, there is no apparent increase, which may be the consequence of the following: the initial carbon content of lc-ZnEp, lc-uc and lc-Ep falls within 800–850 ppm, which is relatively high considering that 1000 ppm is reported to be the approximate limit to the C content around which the coercivity drops dramatically [13,20,21]. Due to the high atomic mass of Nd compared to impurities like O and C, as well as the low amount of active Nd-rich phase in the overall magnet matrix, the effects of seemingly low contamination should not be underestimated.

For hc-Ep, while the initial carbon content is moderate at ~670 ppm, the O content is high at ~2720 ppm. Therefore, both saturation and a considerable variation in values may prevent significant carbon uptake.

Another important factor seems to be the usage of passivation underneath the contemporary epoxy coatings, which likely functions as a barrier against (carbon) diffusion.

In contrast to the presented results, moderate carbon uptake was measured in one specific sample analyzed in a screening experiment before this study, but it could not be appropriately repeated due to a lack of specimens of this sample type. What sets this sample apart was that it was an old, rather outdated magnet design featuring no passivation below the coating.

### 3.4. Reversibility of the Contaminant Uptake

The visible discoloration is shown Figure 1, as well as the SEM-EDS analysis of the surface areas are seen in Figure 2 and Figure 3. Figure 2 shows that the uptake of oxides is very localized in the close-to-surface regions of the magnet. The same is assumed for the hypothetical uptake of carbon. Therefore, experiments were conducted by taking (i) non-demagnetized samples, (ii) demagnetized samples and (iii) demagnetized samples that underwent sandblasting to remove all visible discoloration.

**Figure 7:**
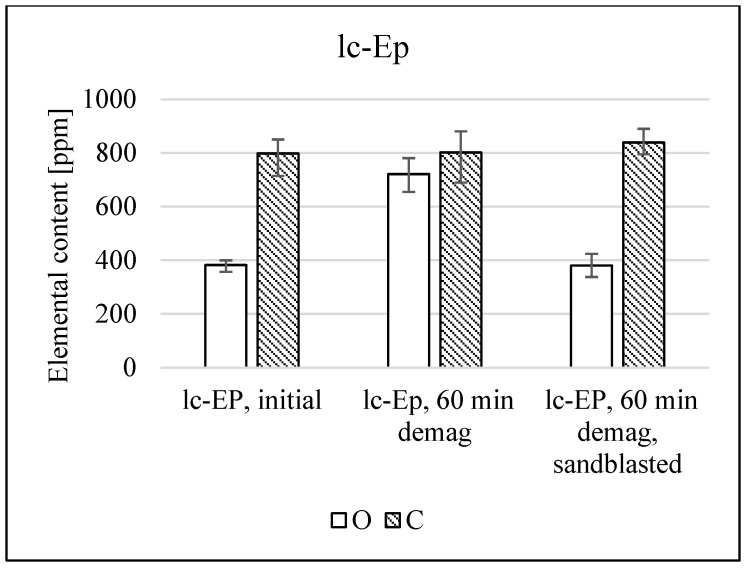
Comparison of the average oxygen and carbon content of lc-Ep before and after demagnetization as well as after the subsequent sandblasting of the darkened surface layer. Measured with Leco ONH836 and CS744.

Sandblasting the darkened layer for 1–2 s per surface until no more changes in surface coloration are visible on average removes 10–25 µm of material from each side (measured on Samples lc-uc). In Figure 7, it is seen that the O values were successfully lowered, approximately reaching the initial values.

The oxygen and carbon content could not be reduced below the initial values before demagnetization. This also applies to samples with very high initial oxygen content, such as hc-uc. As explained previously, these samples are assumed to be saturated with oxides and carbides. They appear to have oxides distributed throughout the material instead of only within a limited depth near the surface.

Therefore, the effect of thermal demagnetization on organic contamination can be successfully mitigated by ensuring that mechanical coating removal for all samples is carried out only after the thermal demagnetization process. This method only works in a limited depth and leads to some material loss.

Coating removal has to be carried out regardless; while Ni-based coatings can be removed from the powder [6,22], Zn coatings, epoxy coatings, glues and oxide layers should be removed to avoid the contamination of the material to be re-sintered.

Zn residues evaporate below the sintering temperature, depositing on and damaging the equipment. Due to their high vapor pressure, high concentrations of Zn may further impair the density and integrity of the magnet.

Under certain conditions, the Zn coating can be removed, despite the infiltration caused by thermal demagnetization. As seen in Figure 3, the Zn infiltration depth increases over time and only reaches a few µm after 60 min but can go up to approximately 150 µm after 240 min. Therefore, a subsequent sandblasting treatment could remove the infiltration if the demagnetization time is kept as short as possible. Regarding Zn infiltration, monitoring the surface color alone does not facilitate the targeted removal of the Zn contamination, as it has similar optical characteristics to non-infiltrated NdFeB material.

The excessive carbon uptake from the remaining carbon-rich contamination will reduce coercivity by forming carbides, reducing the active Nd-rich grain boundary phase [23] and disproportionating the hard magnetic phase [13].

## 4. Conclusions

In this paper, the development of the oxygen and carbon content of samples with variable coatings and initial contamination levels during thermal demagnetization was tested, and it was trialed whether the contamination uptake can be mitigated. A general overview of the samples, sample designations and testing parameters is given in Table 1.

In the following recommendations for the magnets’ design, the thermal demagnetization parameters and the integration of this process into the recycling process are given: Demagnetization times should be as short as possible to minimize the uptake of unwanted contaminants, including O, C and especially Zn. After 15 min at 400 °C, all magnets were fully demagnetized. Longer hold times may be necessary for larger magnets.

Generally, thermal demagnetization with the added objective to facilitate the removal of adhesive-fixed magnets or to split segmented magnets is better employed in air, as it allows faster and more complete decomposition [10]. While demagnetizing in a protective atmosphere may be beneficial to avoid oxygen uptake and preserve coatings, which would be helpful for reuse applications, it is not recommended for recycling. High costs for inert process gas and the required infrastructure to introduce it can be circumvented. The method’s shortcomings, namely the possible contamination with O, C and Zn, can be mitigated by adjusting the order of processes and processing parameters or avoided using suitable coatings. The mechanical removal of coatings should be generalized for all samples without sorting and employed after thermal demagnetization, not before. This order is essential, as the O content increases strongly in uncoated samples.

The demagnetization temperature is best kept low but should be high enough to disintegrate all organic adhesives. Using a hold temperature of 400 °C proved to be sufficient and the adverse effects of the treatment were successfully reversed.

Among the tested coatings, NiCuNi stands out as the most effective in protecting the magnets from oxidation during thermal demagnetization, as demonstrated in Figure 5. It showed the lowest relative oxygen uptake, consistent with NiCuNi’s excellent performance in corrosion tests like the salt spray test [23]. It functions well as a physical barrier to chemical and oxidative attacks. Overall, NiCuNi, as well as other Ni-based coatings, are a promising choice for preserving the chemical properties of magnets during their lifetime as well as during separation for recycling.

Overall, thermal demagnetization in air as a preparative measure before recycling via HPMS and re-sintering, which is a recycling technique with a high sensitivity towards contamination, is viable. For less sensitive methods like hydrometallurgical recycling, where the material can be sufficiently purified as it is first leached and then the metals are extracted [24], it is also viable. It can be regarded as a simple and cost-effective procedure that can be carried out by any recycling facility owning at least a simple furnace operating at up to 400 °C. Due to the formation of hydrocarbon fumes during the decomposition of adhesive and organic coatings, suitable waste gas filtration or treatment must be employed. Due to widespread legislation to avoid air pollution, like the EU-wide Industrial Emissions and Livestock Rearing Emissions Directive (IED) [25] or the US Clean Air Act (CAA) [26], among others, such gas treatment facilities should be available at most recycling facilities.

Therefore, this simple method can be employed without reservation if the abovementioned conditions are met, especially for separating NdFeB from other materials and safely handling scraps.

In a possible, more advanced version of this process, mechanical coating removal could be aided by an optically controlled system to minimize material losses. A system like this could remove any surface layers with a different shine or color than the NdFeB alloy, removing coatings, passivation layers and unwanted oxide layers.

Furthermore, it is acknowledged that there is a lack of published research concerning the effects of contamination originating from various elements beyond those introduced by thermal demagnetization as presented in this paper. Only a few studies have investigated the influence of contaminants on pure rare earths [27], on the properties of NdFeB magnets specifically and on the structural incorporation or substitution of elements in NdFeB [28]. The research and review of these fields should be greatly encouraged.

## Figures and Tables

**Table 1:** Sample classification and designation as well as the testing parameters and condition of the tested samples summarized to facilitate the understanding of the report.

Sample Classification and Designation:	Testing Parameters	
**Initial oxygen content** <1000 ppm → low contamination (lc) >1000 ppm → higher contamination (hc)	**Thermal demagnetization conditions** Temperature: 400 °C Atmosphere: Air	
**Coating/surface type** Uncoated Epoxy-coated (-Ep) Zinc–epoxy-coated (-ZnEp) Zinc-coated (-Zn) NiCuNi-coated (-NiCuni)	**Variation of the condition of tested samples**	
	Treatment Untreated Thermally demagnetized Thermally demagnetized and sandblasted	Hold time 15 min 30 min 60 min 120 min 180 min 240 min

## Data Availability

The data presented in this study are available on request from the corresponding author due to privacy.
